# Prevalence and associated factors of severity levels of anemia among women of reproductive age in sub-Saharan Africa: a multilevel ordinal logistic regression analysis

**DOI:** 10.3389/fpubh.2023.1349174

**Published:** 2024-01-24

**Authors:** Lire Lemma Tirore, Abriham Shiferaw Areba, Aklilu Habte, Mitiku Desalegn, Abraham Sahilemichael Kebede

**Affiliations:** ^1^Department of Public Health, College of Medicine and Health Sciences, Wachemo University, Hossana, Ethiopia; ^2^Department of Anesthesia, College of Medicine and Health Sciences, Wachemo University, Hossana, Ethiopia; ^3^MSCA Fellow, School of Sport and Health Sciences, University of Brighton, Brighton, United Kingdom

**Keywords:** severity levels, anemia, women, multilevel analysis, sub-Saharan Africa

## Abstract

**Background:**

Sub-Saharan Africa is the most anemia-prone region, with several of the sub-region’s countries having a substantial prevalence of the anemia among women of reproductive age. Nonetheless, no adequate study has been conducted to illustrate severity levels and associated factors of anemia among women of reproductive age. Therefore, this study presents the most recent estimates on the prevalence and severity levels of anemia and its associated factors among women of reproductive age in 21 Sub-Saharan Africa countries.

**Methods:**

This study used the most recent Demographic Health Survey (DHS) datasets, which were collected in 21 sub-Saharan African countries between 2015 and 2022. A total of 171,348 women of reproductive age were included in the analysis. Multilevel (three-level) ordinal logistic regression was done to identify factors associated with severity levels of anemia.

**Results:**

The pooled prevalence of anemia was 41.74%. The pooled prevalence of mild, moderate and severe anemia was 23.45, 17.05 and 1.24, respectively. Women who were living at distance to a health facility (AOR = 1.07), women living in the poorest households (AOR = 1.49), women living in the households with unimproved toilet (AOR = 1.12) and in households that were using solid cooking fuel (AOR = 1.10), pregnant women (AOR = 1.72) and those who have given birth to more than one children within 3 years (AOR = 1.43) had greater odds of higher levels of anemia as compared to their counterparts. Women who were in the age groups of 20–24 (AOR = 0.81), 25–29 (AOR = 0.78), 30–34 (AOR = 0.79), 35–39 (AOR = 0.88), and 45–49 (AOR = 0.89), women who have attended primary school (AOR = 0.50), secondary (AOR = 0.57) and higher education (AOR = 0.76) and who were living in rural area (AOR = 1.07) had lower odds of higher levels of anemia as compared to their counterparts.

**Conclusion:**

Considering individual, household and community contexts is necessary while formulating and implementing anemia prevention and control policies. Adolescent women, and women who did not attend education and at a distance to a health facility should get especial attention while implementing anemia prevention and control programs.

## 1 Introduction

Anemia is a disorder when the hemoglobin (Hb) concentration, or the quantity and size of red blood cells, falls below a predetermined threshold, hence reducing the blood’s ability to carry oxygen throughout the body. Anemia is a sign of poor health and inadequate nutrition (1).

Iron deficiency is the leading cause of anemia, accounting for an estimated 50% of cases in women globally, despite the disease’s complex character (2). Intestinal parasitic infections, malaria, chronic illness, menstrual blood loss, gynecological and obstetric conditions, various nutritional deficiencies (particularly in folate and vitamins B12, A, and C), genetic disorders (such as sickle cell disease, thalassemia, and hereditary blood problem), loss of appetite, low dietary diversity score, poor dietary habit, household food insecurity are additional significant causes of anemia (3).

If a woman’s Hb level is less than 12 g/dL for non-pregnant women and less than 11 g/dL for pregnant women, she is anemic (1). Based on Hb level, anemia is categorized as mild, moderate, or severe (4).

Its clinical manifestations and associated complications differ depending on the type of anemia and its severity (3). Anemia impairs women’s health and wellbeing and raises the possibility of unfavorable consequences for mothers and newborns (2). Preterm birth, low birth weight, miscarriages, stillbirths, and other morbidities are among the risks of maternal anemia that affect both the mother and the child (5). It has contributed to over 25% of maternal deaths, newborn mortality, fetal impairment, and infant death (6, 7). Anemia impairs the learning and development of future generations of children as well as the economic productivity and development of communities and countries (5). Each individual with anemia bears an economic burden ranging from $US29, 511 to $US7, 092 (8).

The most difficult nutritional issue women of reproductive age facing worldwide is still anemia, and the overall burden of this condition may increase. The prevalence of anemia in women who are of reproductive age has been stable worldwide since 2000 (9). The prevalence of anemia among women of reproductive age (WRA) accounts for nearly one-third of the worldwide cases (10, 11).

In the world in 2019, anemia affected half a billion (30%) WRA and 37% of pregnant women (12). When it comes to anemia (62.3%) and severe anemia (1.8%), Africa was ranked the second. Moreover, the prevalence of anemia is rising alarmingly in Africa for both pregnant and non-pregnant women, rising from 38.9 to 48.7% and 37.7 to 41.5%, respectively (11). Almost 40% of these women reside in developing regions, including sub-Saharan Africa (SSA) (10, 11). SSA is the most anemia-prone region, with several of the sub-region’s countries having a substantial prevalence of the condition (13, 14). In all of WRA (38.6%) and among pregnant women (54%) in SSA, it is a moderate and severe public health problem, respectively (15).

Factors like age, education, reproductive and obstetric factors, and maternal health service usage and residency have been found to be associated with anemia in WRA (16–19).

Given that anemia affects billions of people globally and puts them at risk for a range of social, psychological, and economic crises, governmental and non-governmental organizations are closely monitoring the issue as a public health concern (5, 20, 21). Aiming to cut the prevalence of anemia in women of reproductive age by 50% by 2025 was approved by the World Health Assembly in 2012 (22). Additionally, by 2030, the global maternal mortality ratio is expected to drop to less than 70 per 100,000 live births, according to the Sustainable Development Goal. At the regional level, trustworthy and efficient data and monitoring systems would be essential to meet these goals (5). In order to develop timely interventions in the prevention and treatment of anemia, it is imperative that sufficient evidence regarding the individual and community-level factors contributing to anemia be generated. However, there are only limited studies estimating the up-to-date pooled prevalence and associated factors of the severity levels of anemia in WRA in SSA. In order to narrow this gap, the current study used the most recent data that were available from the DHS of the 21 SSA countries. Nevertheless, the present study presents the most recent estimates on the prevalence and severity levels of anemia and its associated factors, which will be useful for public health policymakers. This study used multilevel (three-level) ordinal logistic regression to identify factors associated with severity levels of anemia because the DHS data are hierarchical, in which individuals are nested within households, and households are nested within clusters (communities).

Addressing what factors contribute to anemia in SSA women of reproductive age will assist in ensuring mothers and babies have healthier pregnancy outcomes, which will benefit future generations’ health, wellbeing, and potential for economic growth and community development. It is necessary to generate adequate evidence on individual and community-level factors of anemia, which is highly crucial for the development of timely interventions in anemia prevention and treatment (5).

## 2 Methods

### 2.1 Data source and sampling procedure

This study used the most recent Demographic Health Survey (DHS) datasets, which were collected in 21 sub-Saharan African countries between 2015 and 2022. The primary goal of the nationally representative DHS survey is to enhance the gathering, processing, and sharing of population, health, and nutrition data while streamlining their application to planning, policy-making, and program administration (3). We were granted the permission from the Inner City Fund (ICF) International to access the datasets. Then individual data set was downloaded from the DHS website.^1^ Details about the DHS are available in the DHS report of each country.

We have appended the datasets together to estimate the pooled prevalence and identify factors associated with the severity levels of anemia among WRA in sub-Saharan Africa.

**Figure 1:**
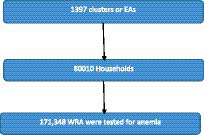
Sampling procedure for selecting the women of reproductive age for DHS survey.

Two-stage stratified sampling technique was applied. First, each region of the country was stratified into urban and rural areas. Then, random samples of Enumeration Areas (EAs) were selected from urban and rural area independently. In the second stage of selection, a fixed number of 20 to 28 households per EA were selected with an equal probability systematic selection. All WRA in the selected HH were eligible for anemia testing. Then, hemoglobin testing was carried out among voluntarily consented WRA in the selected households using HemoCue rapid testing methodology. For the test, a drop of capillary blood was taken from a women’s fingertip and was drawn into the micro cuvette which was then analyzed using the photometer that displays the hemoglobin concentration. Then, anemic status was determined based on the hemoglobin level. Hb levels were adjusted for pregnancy because during pregnancy the increase in maternal blood volume and the iron needs of the fetus decrease the blood Hb level. It was also adjusted for smoking and altitude (Figure 1).

The World Health Organization (WHO) Hb cut off points for diagnosis of anemia are given in Table 1.

**Table 1:** World Health Organization (WHO) Hb cut off points for diagnosis of anemia (1).

Hemoglobin cutoff point	Anemia category			
	No anemia	Mild	Moderate	Severe
For pregnant women	>11	10.0–10.9	7.0–9.9	<7.0
For non-pregnant women	>12	10.0–11.9	7.0–9.9	<7.0

The individual record dataset was used in this study. The study included all WRA with anemia status data. A total of 171,348 WRA from 21 SSA countries who were tested for anemia were included in the analysis. Using the STATA software, we extracted the independent and dependent variables for every nation and then appended the data. Data are gathered for DHS surveys using four main questionnaires. Data regarding the characteristics of the household are gathered using the Household Questionnaire. The Biomarker Questionnaire gathers data on hemoglobin levels and anthropometric measurements. Women and men are interviewed using the Woman’s Questionnaire and the Man’s Questionnaire, respectively. The data from a DHS survey forms a hierarchy of households within a cluster and household members within each household (3).

### 2.2 Dependent variable

Anemia is an ordinal dependent variable and is categorized as none, mild, moderate, or severe anemia based on Hb level.

#### 2.2.1 Independent variables

Independent variables were considered based on their availability in the dataset, in the literature, and their theoretical and practical significance. Due to the hierarchical nature of the DHS dataset, the independent variables were classified as individual, household, and community-level variables.

Individual-level variables were characteristics of the women, which were specific to each woman. These are age, educational status, marital status, mass media exposure, smoking cigarettes or tobacco, age at first birth, number of births in the last 3 years, and pregnancy. Household-level variables are those variables whose values are the same for all women living in the same household. These are the number of household members, wealth index, toilet facility, water source, time to water source, distance to health facility, and type of cooking fuel. Community-level variables are those variables whose values are the same for all women living in that community. These are the place of residence and region of residence.

### 2.3 Statistical analysis

The data were cleaned and analyzed using STATA version 14. The data were summarized using proportions, means, and medians. Tables and graphs were used to organize the data. Multilevel (three-level) ordinal logistic regression was done to identify factors associated with severity levels of anemia. This is because the DHS data are hierarchical, in which individuals are nested within households, and households are nested within clusters (communities).

### 2.4 Model specification

The multilevel (three-level) ordinal logistic regression model is given as follows:


$$\log\left[\frac{\mathrm{Pijkc}\;}{1-\mathrm{Pijkc}}\right]=c-\left(\mathrm{xijk\beta}+uij+ui\right)$$

Where:

P_ijkc_—is accumulative probability of being at “c” category of anemia for kth individual in jth household and ith cluster.

C—is a model threshold or intercept for C-1 level of anemia, and it is a fixed parameter.

C = number of categories of anemia.

Β—is a coefficient (fixed effect of explanatory variable).

X_ijk_—is a covariate vector for kth individual in jth household and ith cluster.

uij—is level-2 (household) random effect.

ui —is level-3 (cluster) random effect.

Five different models were fitted.

The first model was the null model, which is a model with no independent variables. It showed the variance in anemia among women of reproductive age attributed to the household and cluster differences in the absence of the explanatory variables.

The second model was a model with individual-level variables. The third and fourth models were models with household and community-level variables, respectively. The fifth model was a model containing significant variables from the 2nd, 3rd, and 4th models.

The variance partition coefficient (VPC) and proportional change in variance (PCV) were used to quantify the random effects of households and clusters. The VPC measures the percentage of variation in the outcome (anemia) that cannot be explained by the predictor variables at each level of the model hierarchy (23). It measures the relative significance of clusters, households and individual (women) as sources of variation on anemia status. PCV measures the explained variances by the model’s variables at the cluster and household levels (24).

The STATA command “meologit” was used to fit these models. Considering the complex sampling design of the survey, a sample weight was applied. The adjusted odds ratio was estimated with a 95% confidence interval. Variables with a *p* < 0.05 in each model were considered for the full model. The proportional odds assumption was tested using the likelihood ratio test, and the assumption was satisfied (*p* = 0.67).

Models were compared using Akaike information criteria (AIC), and the model with the smallest AIC was considered the best fit.

### 2.5 Ethics approval

Permission to use the DHS data was obtained from Inner City Fund (ICF) International after registering and stating the goal of the study. During the DHS data collection, ethical clearance was obtained from the Ethics Committee of ORC Macro Inc.

## 3 Results

### 3.1 Individual level characteristics of the participants

The median age of the respondents was 28.60 years (SD = 9.55 years). More than one-fifth (21.52%) were in the age group of 15–19 years, and around one-third (33.53%) have attended secondary school. The average age at first birth was 19.40 years (SD = 3.85). Majority of them (91.94%) were not pregnant, 63.24% were living with partner and 33.23% had one birth in last 3 years (Table 2).

**Table 2:** Frequency and percentage distribution of individual level characteristics of WRA in SSA, 2023.

Variables	Category	Frequency	Percent
Age in 5-year groups	15–19	37,055	21.63
	20–24	31,222	18.22
	25–29	28,558	16.67
	30–34	24,384	14.23
	35–39	21,193	12.37
	40–44	15,997	9.34
	45–49	12,939	7.55
Women educational status	No education	50,949	29.73
	Primary	55,034	32.12
	Secondary	57,163	33.36
	Higher	8,202	4.79
Pregnancy	No	157,250	91.77
	Yes	14,098	8.23
	One	27,578	16.09
	More than one	584	0.34
Births in	No 103,549	60.43	60.43
Last three	One	59,619	34.79
Years	More than one	8,180	4.77
Age at first birth	<18	42,471	34.09
	≥18	82,126	65.91
Marital status	Living with partner	107,181	62.55
	Not living with partner	64,167	37.45
Mass media exposure	Not at all	53,705	31.34
	Less than once/week	30,934	18.05
	At least once/week	67,540	39.42
	Everyday	19,169	11.19
Smoking cigarette or tobacco	No	169,647	99.01
	Yes	1,701	0.99

Source: DHS report of each country.

### 3.2 Household level characteristics of the participants

More than half (54.60%) of the women were living in households with improved toilet facility and more than three-fourth of them (76.10%) were living in households with improved water source. Around one-thirds (32.74%) of the women had drinking water on premises. More than three-fourth (87.68%) of the women were living in households which uses clean cooking fuel and nearly one-fifth (20.25%) of the women were living in poorest households. The average number of household member the women were living with was 6.57 (SD = 4.23) (Table 3).

**Table 3:** Frequency and percentage distribution of household level characteristics of WRA in SSA, 2023.

Variables	Category	Frequency	Percent
Type of toilet	Unimproved	78,53	45.83
	Improved	92,814	54.17
Water source	Unimproved	41,593	23.90
	Improved	132,379	76.10
Type of cooking fuel	Clean	150,212	87.66
	Solid	21,136	12.34
Number of household members	≤2	11,710	6.83
	3 or 4	41,765	24.37
	≥5	117,873	68.79
Wealth index	Poorest	34,230	19.98
	Poorer	31,495	18.38
	Middle	32,864	19.18
	Richer	34,100	19.90
	Richest	38,659	22.56
Time to water source	≤30 min	84,509	49.32
	More than 30 min	34,032	19.86
	On premises	52,807	30.82
Distance to health facility	Big problem	63,001	36.77
	Not a big problem	108,347	63.23

### 3.3 Community level characteristics of the participants

More than half (52.18%) of the women were from East Africa and 38.61% were from West Africa. More than half of them (62.47%) were rural residents (Table 4).

**Table 4:** Frequency and percentage distribution of community level characteristics of WRA in SSA, 2023.

Variable	Category	Frequency	Percentage
Region of residence	West Africa	66,152	38.61
	Burkina Faso	8,765	5.12
	Benin	8,011	4.68
	Gambia	5,914	3.45
	Mali	5,090	2.97
	Nigeria	14,750	8.61
	Liberia	4,065	2.37
	Côte d’Ivoire	7,085	4.13
	Guianea	5,262	3.07
	**Central Africa**	**12,814**	**7.48**
	Cameroon	6,809	3.97
	Gabon	6,005	3.50
	**East Africa**	**89,408**	**52.18**
	Ethiopia	14,489	8.46
	Burundi	8,539	4.98
	Madagaskar	9,487	5.54
	Malawi	7,970	4.65
	Ruwanda	7,299	4.26
	Sera Leone	7,210	4.21
	Tanzania	13,102	7.65
	Uganda	6,031	3.52
	Zambia	13,226	7.72
	Zimbabwe	9,265	5.41
	**South Africa**		
	Republic of South Africa	2,974	1.74
Place of residence	Urban	64,303	37.53
	Rural	107,045	62.47

The bold values show the region of the countries listed below them.

### 3.4 Prevalence of anemia in WRA in SSA

**Figure 2:**
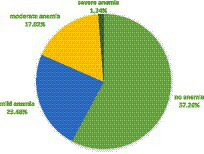
The pooled prevalence of severity levels of anemia among WRA in SSA, 2023.

The mean hemoglobin was 12.09 g/dL (SD = 1.731 g/dL). The pooled prevalence of anemia was 41.74%. More than one in 10 (1.24%) and 17.02% of the women were severely and moderately anemic, respectively (Figure 2).

### 3.5 Random effects

The null model showed that more than a quarter [VPC (2 + 3) = 27.51%] of the total variation in anemia was due to unobserved household and community level factors, and 25.20% [VPC (2) = 25.20%] of the total variation in anemia was due to unobserved household level factors. The PCV_3_ indicated that 35.236% of the variation in anemia between communities was explained by individual, household and community-level characteristics (Table 5).

**Table 5:** Random intercept variances of three-level mixed effects models, 2023.

Random effects	Model 1	Model 2	Model 3	Model 4	Full model
σ^2^ (ν3)	0.105	0.133	0.131	0.065	0.068
σ^2^ (ν2)	1.144	1.138	1.160	1.139	1.585
VPC_(2 + 3)_ (%)	27.51	26.46	28.18	26.78	33.44
VPC_(2)_ (%)	25.20	23.69	25.32	25.34	32.06
VPC_(3)_ (%)	2.31	2.76	2.85	1.44	1.37
PCV_3_	Reference			23.52	35.23

NB: σ2 (ν3) and σ2 (ν2) are community and household random intercept variances, respectively. VPC (3), variance partition coefficient of cluster, VPC (2 + 3), variance partition coefficient of household and cluster, VPC (2), variance partition coefficient of household, PCV_3,_ proportional change in variance.

### 3.6 Factors associated with anemia among WRA in SSA

Model 5 (the model adjusted for individual, household, and community-level variables) was considered a best-fit model because of the smallest AIC (321557.4) (Table 6).

**Table 6:** Factors associated with severity levels of anemia among WRA in SSA, 2023.

Variables	Model 2 AOR (95% CI)	Mode 3 AOR (95% CI)	Model 4 AOR (95% CI)	Model 5 AOR (95% CI) (final model)
Age				
15–19	1			1
20–24	0.83 (0.80, 0.86) *			0.81 (0.76, 0.87)*
25–29	0.76 (0.73, 0.79) *			0.78 (0.73, 0.84)*
30–34	0.76 (0.73, 0.79) *			0.79 (0.73, 0.85)*
35–39	0.84 (0.80, 0.86) *			0.88 (0.81, 0.95)*
40–44	0.90 (0.84, 0.94) *			0.96 (0.89, 1.04)
45–49	0.86 (0.84, 0.96) *			0.89 (0.81, 0.97)*
Educational status				
No education	1			1
Primary	0.58 (0.50, 0.78)*			0.50 (0.48, 0.51)*
Secondary	0.55 (0.81, 0.96)*			0.57 (0.57, 0.60)*
Higher	0.56 (0.51, 0.98)*			0.76 (0.71, 0.81)*
Pregnancy				
No				1
Yes	1.75 (1.62, 1.82)*			1.72 (1.63, 1.81)*
Births in last 3 years				
No	1			1
One	1.12 (1.09, 1.15)*			1.04 (0.99, 1.09)
More than one	1.43 (1.35, 1.51)*			1.16 (1.07, 1.26)*
Marital status				
Living with partner				1
Not living with partner	1.04 (1.01, 1.07)*			1.19 (0.15, 1.24)
Mass media exposure				
Not at all	1.01 (0.86, 1.06)			
Less than once/week	1.01 (0.97, 1.07)			
At least once/week	1.00 (0.96, 1.05)			
Everyday	1			
Smoking cigarette or tobacco				
No				
Yes	1.00 (0.89, 1.13)			
Type of toilet				
Unimproved		1.23 (1.02, 1.56)*		1.07 (1.04, 1.11)*
Improved		1		1
Water source				
Unimproved		1		1
Improved		1.03 (1.00, 1.06)*		0.97 (0.94, 1.00)
Type of cooking fuel				
Clean		1		1
Solid		1.66 (1.60, 1.72)*		1.10 (1.05, 1.16)*
Number of household members				
≤2		1		
3–4		0.92 (0.87, 0.96)*		0.90 (0.84, 1.97)
≥5		1.09 (0.04, 1.14)*		0.92 (0.86, 1.99)
Wealth index				
Poorest		1.94 (1.85, 2.04)*		1.49 (1.49, 1.57)*
Poorer		1.64 (1.57, 1.72)*		1.31 (1.25, 1.38)*
Middle		1.50 (1.44, 1.56)*		1.25 (1.19, 1.31)*
Richer		1.28 (1.23, 1.33)*		1.12 (1.08, 1.17)*
Richest		1		1
Time to water source				
≤30 min		0.74 (0.21, 0.85)*		0.91 (0.88, 1.95)
>30 min		0.52 (0.41, 0.79)*		0.84 (0.80, 1.88)
On promises		1		1
Distance to health facility				
Big problem		1.05 (1.03,1.08)*		1.07 (1.04, 1.09)*
Not a big problem		1		1
Place of residence				
Urban			1.18 (1.15, 1.21)*	1.07 (1.03, 1.12)*
Rural			1	1
Region of residence				
West Africa			3.89 (3.78, 3.99)*	3.45 (3.34, 3.56)*
Central Africa			3.71 (3.53,3.90)*	3.31 (3.14, 3.48)*
South Africa			1.09 (0.99, 1.21)	1.03 (0.93, 1.14)
East Africa			1	1
Model fit statistics				
AIC	330918.9	332028.1	323089.7	321557.4

*p < 0.05.

As the full model showed, age, education, number of births in the last 3 years, pregnancy, toilet, distance to a health facility, cooking fuel, wealth index, place, and region of residence were significantly associated with anemia.

Those women who were in the age groups of 20–24, 25–29, 30–34, 35–39, and 45–49 had 19% (AOR = 0.81), 22% (AOR = 0.78), 21% (AOR = 0.79), 12% (AOR = 0.88), and 11% (AOR = 0.89) lesser odds of being at higher levels of anemia as compared to those women in the age group of 15–19 years, respectively.

Women who have attended primary school, secondary and higher education were 50% (AOR = 0.50) 43% (AOR = 0.57) and 24%(AOR = 0.76) less likely to be at higher levels of anemia, respectively, as compared to women who have not attended education. Pregnant women were 1.72 (AOR = 1.72) times more likely to be at higher levels of anemia as compared to non-pregnant women. The odds of being at higher levels of anemia were 1.12 (AOR = 1.12) and 1.43 (AOR = 1.43), respectively, for women who gave birth to one child and more than one child, respectively, as compared with women who gave no birth. The odds of being at higher levels of anemia were 1.07 times greater for women living in households with unimproved toilets as compared to women living in households with improved toilets.

Women who were living in households that were using solid cooking fuel had 10% (AOR = 1.10) greater odds of being a higher level of anemia as compared to women living in households that were using clean cooking fuel. The odds of worse anemia were 49% (AOR = 1.49), 31% (AOR = 1.31), 25% (AOR = 1.25), and 12% (AOR = 1.12) higher for women who were living in the poorest, poorer, middle, and rich households, respectively, as compared to women who were living in the richest households. The odds of being at higher levels of anemia were 1.07 (AOR = 1.07) times greater for women for whom the distance to a health facility is a big problem as compared to women for whom the distance to a health facility is not a big problem. Women who were living in urban areas had 1.07 (AOR = 1.07) times greater odds of being at higher levels of anemia as compared to rural women. Women who were living in West and Central Africa had 3.45 (AOR = 3.45) and 3.31 (AOR = 3.31) times greater odds of being at higher levels of anemia as compared to women living in East Africa (Table 6).

## 4 Discussion

The pooled prevalence of Anemia in SSA (41.74%.) is higher than the global average of 29.9%. The likelihood that a woman would have greater levels of anemia was lower in the age categories of 20–24, 25–29, 30–34, 35–39, and 45–49 than in the 15–19 age group. The results from Ethiopia (25), Nepal (26), and China (27) are consistent with this. The increased iron requirement brought on by adolescent growth may be the cause of this. Women require more iron and other minerals to maintain their rapid growth during their adolescent years. Their vulnerability to anemia is a result of these (28, 29).

Compared to women who have not attended school, those who have are less likely to have greater levels of anemia. Research conducted in sub-Saharan Africa (25), Nepal (26), and Mali (30), supports this conclusion. This could be because they learned about nutrition during their school years. Women with higher levels of education are better able to access household resources that are crucial to their nutritional status, are more knowledgeable of how to use the resources at their disposal to enhance their nutritional status, and are capable of making independent decisions about nutrition. Additionally, there is proof that dietary interventions in schools might enhance students’ knowledge and perspectives on anemia and how to prevent it (31). A Gambia study found no correlation between anemia and education level. The disparities in the variables taken into consideration may be the cause of this disagreement. Variables pertaining to service consumption, such as the use of iron and prenatal care, were taken into account in the Gambia study. Compared to educational status, these variables are more proximal and can explain anemia (32).

Compared to non-pregnant women, pregnant women were more likely to have greater levels of anemia. Studies conducted in the Gambia (32), Mali (30), and China (27) revealed similar results. Pregnant women may be more susceptible to anemia due to the increased iron requirements for both the mother and the fetus. Their body uses iron during pregnancy to produce extra blood, which gives their unborn child oxygen. Additionally, the volume of blood increases during pregnancy. Because more iron and vitamins are required to produce more red blood cells, this lowers hemoglobin (Hb) levels physiologically (33, 34).

Women who gave birth to one child or more within 3 years were more likely than those who gave birth to none at all to have higher levels of anemia. Studies conducted in Ethiopia (35) and China (27) found consistent results. This could be as a result of the higher risk of blood loss associated with childbirth during every pregnancy. Additionally, there’s a higher chance of prenatal and postpartum hemorrhage for them. Short birth interval women may also find it difficult to replenish iron and other micronutrients lost after delivery (36). A Gambia study found no association between anemia and the number of births within 3 years (32). Considering the number of childbirth as continues variables in study done in Gambia could explain the disparity between studies.

Women who felt that distance to a health facility was a big problem were more likely to have higher levels of anemia than women who did not think that distance was a big problem. This aligns with research results from the Gambia East Africa (37) and Gambia (32). The observed outcome may be explained by the inaccessibility of treatments for disorders that cause anemia due to distance from health facilities, as well as the inaccessibility of maternal health services including prenatal and postnatal care. It may be difficult for women who live far from the health facility to get preventive and therapeutic care.

In comparison to women living in households with improved toilet facilities, those living in households with unimproved toilet facilities had greater odds of being at higher levels of anemia. Results from East Africa from East Africa (37), Lao (38), and SSA (25) are in line with this. This might be because women living in an unhygienic environment are at increased risk of food and waterborne diseases like hookworm and diarrheal diseases, which cause anemia. Contaminated water sources are more common in families with unimproved toilets. Gastrointestinal infections can occur as a result of drinking water tainted with bacteria, parasites, or other illnesses. These infections, especially the chronic ones, can lead to nutrient malabsorption, including iron, which is necessary to avoid anemia (39).

Compared to women living in the richest households, the odds of having worse anemia were higher for women living in the poorest, middle, richest, and poorest households, respectively. These results were also seen in studies conducted in Mali (30), East Africa (37), and SSA (25). This might be because women from households with lower wealth index cannot afford diversified and iron-rich food items, and preventive and curative health services. When they fail to afford the treatment for diseases, they might develop anemia as a complication (40, 41). Also women living in households with lower wealth index are at risk of malnutrition and infectious diseases which cause anemia (42, 43). This might be because women from households with a lower wealth index cannot afford diversified and iron-rich food items and preventive and curative health services. When they fail to afford the treatment for diseases, they might develop anemia as a complication (35, 36). Also, women living in households with a lower wealth index are at risk of malnutrition and infectious diseases that cause anemia (37, 38).

Compared to women living in households utilizing clean cooking fuel, those using solid cooking fuel had an increased chance of having a higher level of anemia. Research conducted in Ethiopia (44), Sri Lanka (45), and China (46) corroborate this. This might be the result of the pollutants in solid fuel causing systemic inflammation. Systemic inflammation reduces red blood cell formation and iron balance, which results in low serum iron levels and anemia. Also, indoor air pollution from solid fuel combustion can lead to higher levels of carbon monoxide which can inhibit the absorption of iron in the gastrointestinal tract. The inhibited iron absorption can contribute to iron-deficiency anemia (47, 48).

Rural women were less likely than urban women to have greater levels of anemia, which is in line with research from East Africa (37) and SSA (25, 49). The lower odds of anemia among rural women may be explained by their greater access to foods high in iron, such as dairy products, eggs, and leafy green vegetables, as a result of their involvement in agriculture. Rural women may have more access to fresh and locally grown products, which can contribute to a more diverse and nutritious diet, reducing the risk of anemia (50).

## 5 Limitations

The observed finding may have been influenced by lack of data on some proximal contributors to anemia, such as dietary diversity, HIV, malaria, and parasite infection. Determining temporal relation is further challenging due to the survey’s cross-sectional design.

### 5.1 Areas for further research

Further researches considering variables like dietary diversity and infections like malaria, HIV, parasite infection, other chronic diseases is warranted.

## 6 Conclusion

Anemia is a sever public health problem among WRA in SSA with a pooled prevalence of 41.74%. The pooled prevalence of mild, moderate and severe anemia was 23.45, 17.05, and 1.24, respectively. Factors affecting anemia evolve at individual, household and community levels.

35.236% of the variation in anemia between communities was explained by individual, household and community-level characteristics. Age, education, number of births in the last 3 years, pregnancy, toilet, and distance to a health facility, cooking fuel, wealth index, place, and region of residence were significantly associated with anemia.

Considering individual, household and community contexts is necessary while formulating and implementing anemia prevention and control policies. Adolescent women, and women who did not attend education and at a distance to a health facility should get especial attention while implementing anemia prevention and control programs. Women empowerment in education and economically should be integrated in anemia prevention and control programs. Improving access to health facility and clean cooking fuel should be considered as anemia prevention strategy. Environmental hygiene and Improving sanitation facilities can help reduce the prevalence of anemia among women living in households with unimproved toilets. Promoting cleaner cooking technologies and raising awareness about the health risks associated with the use of solid cooking fuels should be considered. Sustainable solutions that consider cultural practices, economic realities, and access to alternative technologies are crucial for addressing the complex factors contributing to anemia in these contexts.

## Data availability statement

Publicly available datasets were analyzed in this study. This data can be found at: http://dhsprogram.com.

## Author contributions

LT: Conceptualization, Data curation, Software, Writing – original draft, Writing – review & editing. AA: Conceptualization, Methodology, Writing – review & editing. AH: Software, Validation, Writing – review & editing. MD: Software, Writing – review & editing. AK: Software, Writing – review & editing.

## Footnotes

^1^ http://dhsprogram.com
